# An audit tool for longitudinal assessment of the health-related characteristics of urban neighborhoods: implementation methods and reliability results

**DOI:** 10.1186/s12889-020-09424-8

**Published:** 2020-10-07

**Authors:** Madhumita Ghosh-Dastidar, Gerald P. Hunter, Jennifer C. Sloan, Rebecca L. Collins, Andrea S. Richardson, Wendy Troxel, Natalie Colabianchi, Tamara Dubowitz

**Affiliations:** 1 RAND Corporation Santa Monica, 1776 Main St, Santa Monica, CA 90401 USA; 2 RAND Corporation Pittsburgh, 4570 Fifth Ave #600, Pittsburgh, PA 15213 USA; 3 The Pittsburgh Foundation, Five PPG Place, Suite 250, Pittsburgh, PA 15222 USA; 4 University of Michigan, 1402 Washington Heights, Ann Arbor, MI 48109-2013 USA

**Keywords:** Audit tool, Built environment, Direct observation, Longitudinal assessment, Neighborhood environment, Reliability

## Abstract

**Background:**

Improving the neighborhood environment may help address chronic disease and mortality. To identify neighborhood features that are predictors of health, objective assessments of the environment are used. Multiple studies have reported on cross-sectional assessments of health-related neighborhood features using direct observation. As study designs expand to better understand causation and predictors of change, there is a need to test whether direct observation methods are adequate for longitudinal assessment. To our knowledge, this is the first study to report on the reliability of repeated measurements of the neighborhood environment, and their stability, over time.

**Methods:**

The Pittsburgh Hill/Homewood Research on Neighborhood Change and Health (PHRESH) study conducted longitudinal assessments in two low-income, African American neighborhoods at three waves (years 2012, 2015, 2017). The PHRESH audit tool is a modification of earlier validated tools, with an emphasis on environment features relevant for physical activity, sleep, and obesogenic behaviors. Trained data-collector pairs conducted direct observations of a 25% sample of street segments in each neighborhood. At each wave, we audited a sub-sample of street segments twice and assessed reliability using percentage inter-observer agreement and krippendorf’s alpha statistics. Stability of these items was assessed as exhibiting moderate or high agreement at every time point.

**Results:**

Across waves, a majority (81%) of the items consistently demonstrated moderate to high agreement except for items such as public/communal space, amount of shade, sidewalk features, number of traffic lanes, garden/flower bed/planter, art/statue/monument, amount of trash, and physical disorder. The list of items with poor agreement includes features that are easy to miss (e.g. flower bed/planter), hard to assess from outside (e.g. public/communal space), or may change quickly (e.g. amount of trash).

**Conclusion:**

In this paper, we have described implementation methods, reliability results and lessons learned to inform future studies of change. We found the use of consistent methods allowed us to conduct reliable, replicable longitudinal assessments of the environment. Items that did not exhibit stability are less useful for detecting real change over time. Overall, the PHRESH direct observation tool is an effective and practical instrument to detect change in the neighborhood environment.

## Background

Neighborhoods are important for health [1–4]. In fact, the neighborhood environment has been linked to multiple health outcomes including sleep, mental health, cardiovascular risk, and mortality [5–9]. Certain features (e.g. sidewalks) may directly encourage active transportation and physical activity [10–15] and others (e.g. street lighting, noise) may impact sleep [16–18], which, in turn, may influence chronic diseases [19, 20]. Residents of low-income and racially/ethnically segregated neighborhoods share a disproportionate burden of chronic disease [21], as well as limited access to resources, which could contribute to poor health [22–24]. Improving the neighborhood environment holds promise for addressing health-related behaviors associated with chronic disease and mortality [25].

Micro or granular features of the neighborhood (e.g. street lighting) may affect residents’ experiences more directly than macro-level features (e.g. residential density), thus providing stronger links with health behaviors [26–28]. Also, micro-level features are more easily modified than macro-level features. For example, it takes less time and money to repair a sidewalk than to change the land-use mix of a community. While there are multiple approaches for collecting detailed assessments of micro-features of neighborhoods [29–34], direct observation using audit tools is the preferred approach because it allows for systematic observation of detailed or granular features [27]. Google Street View (GSV) has been increasingly used to observe the built environment and provides a cheaper alternative to direct observation (Clarke et al., 2010; Taylor et al., 2011) [35, 36]. While GSV has demonstrated reliability when assessing certain features of the environment (including types of land use, slope, cycling lane or gathering places), it has certain limitations. Its reliability was not as high when considering detailed features, such as the presence of litter or vacant dwellings, and when making qualitative observations such as the quality of sidewalk or housing (Clarke et al., 2010) [35]. Also, GSV imagery is not available for every street in the U.S. and is updated irregularly (Clarke et al., 2010) [35]. Mixed findings regarding the relationship between micro features of the environment and health outcomes could be due to differences in measurement approaches across studies. An increased interest in the *local* environment for public policy has led to increased emphasis on the rigorous development, implementation and validation of audit tools for direct observation.

In a comprehensive review, Brownson et al. (2009) [27], described multiple audit tools for direct observation of the physical environment [27]. These tools shared some common content including one or more measures of: land use (e.g., presence and type of housing); streets and traffic (e.g., traffic volume); sidewalks; bicycling facilities; public space/amenities (e.g., presence of benches); architecture or building characteristics (e.g., building height); parking and driveways (e.g., parking garage); maintenance (e.g., litter); and indicators of safety (e.g., graffiti). Other features less consistently assessed are noise levels, or health promotion supports (e.g., billboards promoting physical activity) [27]. Existing audit tools have been used for one-time examinations of the neighborhood environment. As designs expand to better understand causation and predictors of change, there is a need to test whether audit tools are adequate for longitudinal assessment.

The Pittsburgh Hill/Homewood Research on Neighborhood Change and Health (PHRESH) study leverages a natural experiment design, comparing an intervention and a control neighborhood, to evaluate whether neighborhood improvements benefit residents’ health [8, 24, 37]. Between 2011 and 2018, the intervention neighborhood received about $200 million, while the comparison neighborhood received approximately $48 million, in publicly-funded investments. Efforts involved physical infrastructure modification (i.e., street lengths, street names, traffic patterns) and construction of streets, housing and landscaping. To systematically document change, we conducted multiple direct observations of the neighborhood environment over a 5-year period with an emphasis on features that may impact physical activity or sleep.

Of the existing audit tools, four were comparable to ours with respect to detail, content and data collection approach: Systematic Pedestrian and Cycling Environmental Scan (SPACES) [38]; St. Louis Analytic Audit Tool and Checklist (SLU) [39, 40]; Systematic Social Observation protocol [29] and Pedestrian Environment Data Scan (PEDS) [41]. Two of these studies reported that 70% of items had kappa statistics [42] above .40, one reported average reliability of .87, while the fourth study reported high inter-observer agreement of 75% or greater [27]. Longitudinal studies may encounter pitfalls if these audit tools are not reliable over time. Mismeasurement can obscure meaningful differences, while systematic bias can produce spurious findings. In this paper, we describe the implementation methods, lessons learned, and stability of reliability estimates from PHRESH longitudinal assessments of the neighborhood environment at three time points over a five-year period. Our findings can help inform future studies of changes in  the built and social environment.

## Methods

### Context

PHRESH is an ongoing study of two low-income and predominantly African American communities in Pittsburgh, PA chosen because of their similarities. Hill District is approximately 1.37 mile^2^ with population of approximately 10,000; while Homewood is 1.45 mile^2^ with population of approximately 8000. Both are residential neighborhoods. We were examining features of the built and social environment that correlate with health, as well as documenting to what extent changes impact residents’ health and well-being, diet, exercise, sleep, heart, and cognitive health. The PHRESH study follows a cohort of individuals and their surrounding physical and social environment to evaluate these questions. Details of the study design have been described elsewhere [43, 44]. To systematically measure change, we conducted assessments of the environment at three timepoints (2012, 2015 and 2017). We modified the Bridging the Gap/Community Obesity Measures Project (BTG-COMP) Street Segment Observation form [45–47], which draws from validated instruments used by other major studies assessing neighborhood features correlated with walking and physical activity [38, 40, 41, 48–50]. All study protocols were approved by the organization’s Institutional Review Board.

### Audit tool

The PHRESH Street Segment Audit (SSA) tool is a detailed assessment of neighborhood-level physical and social features related to health behaviors, with an emphasis on physical activity and sleep. As seen in Table 1, our tool includes (i) Land use mix capturing diversity of land use, (ii) Physical activity (PA) facility to include spaces for play or physical activity; (iii) Walking/cycling environment including presence of sidewalks, shoulders and bike lanes; (iv) Safety signs including traffic calming and control features; (iv) Amenities and litter including features that make a segment appealing and pedestrian friendly, as well as two subjective assessments (perceived safety of walking; perceived attractiveness for walking) to complement the objective assessments. To the existing BTG-COMP audit tool, we added Environment (e.g. trees, cliffs/ravines) and Gathering places (e.g. restaurants, barbershop, church). In the last data collection round (2017), we also added Social disorder items (e.g. presence of police, people selling illegal drugs); a single item on Noise pollution and Physical disorder items (e.g. amount of beer or liquor bottles, abandoned cars), as they have been shown to be related to health behaviors such as sleep [51–53]. See Supplemental Table 1 for a full list of items.

**Table 1:** PHRESH Street Segment Audit (SSA) Items

Audit tool section	Number of items	Sample items
Land use mix	14	Housing, public/civic, office, retail, recreational, vacant.
Environment	6	Slope, slight or steep hill, number of trees, shade from trees, bars on or broken/boarded windows
Physical activity facility	8	Indoor facility, park, playing field, playing court, trail
Walking/cycling environment	20	Street type, vehicular lanes, traffic features, bike lanes, sidewalks
Safety signs	5	Bicycle or pedestrian crossing, kids at play, special speed limit
Amenities and litter	16	Neighborhood or community sign, garden/flower bed/planter, art/statue/monument, benches, drinking fountains, bus stops, trash, perceived safety while walking, attractiveness of street segment for walking
Gathering places	11	Restaurants, libraries, barbershops, churches, bars, corner stores
Social disorder	8	Presence of police or security guard, adults loitering, loud music, people smoking
Noise pollution	1	Level of noise pollution
Physical disorder	8	Broken bottles, drug paraphernalia, graffiti, broken windows

### Street segment selection

The two neighborhoods are residential with almost no arterial segments. Due to homogeneity among street segments within a concentrated geographic area and to reduce costs, we audited a random, representative sample from each of the study neighborhoods. To draw a representative sample, we constructed a complete listing (*n* = 2027) including all segments within a quarter mile of the neighborhood boundaries. The listing was compiled using a geographic shapefile provided by ESRI (ESRI, 2011), and was supplemented with street network information provided by city of Pittsburgh’s GIS department, Google Maps, and personal inspection. The decision to draw a random 25% sample was informed by an earlier published study [54]. Therefore, 511, 585 and 586 segments were sampled in 2012, 2015 and 2017, respectively.

**Fig. 1:**
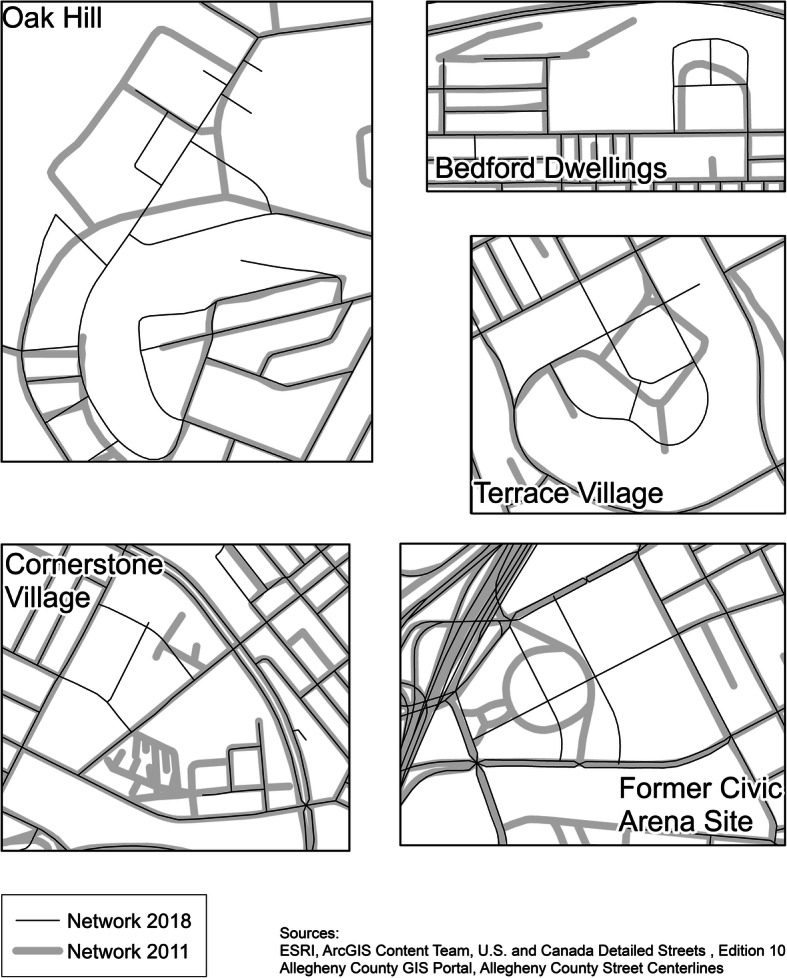
Changing Street Networks. Source: ESRI, ArcGIS Content Team, U.S. and Canada Detailed Streets, Edition 10 Allegheny County GIS Portal, Allegheny County Street Centerlines

Whenever possible, a street segment was followed over time. The planned change in the study neighborhoods affected the nature and existence of some streets. We saw significant changes in areas with public housing (often old, dating back decades). Between 2011 and 2018, $136.5 million and $54.3 million in residential development (including some HOPE VI grants) came into the Hill District and Homewood, respectively. In and around public housing, entire street blocks were demolished; in certain areas, the street grids themselves changed. There were about five areas where street networks themselves changed (not just the buildings on the streets), with the changes shown in Fig. 1. Thus, we had to establish consistent rules to address such changes. Specifically, if a sampled segment did not exist at a follow up wave, a randomly selected segment from the same neighborhood served as replacement. If a sampled segment was bisected, both parts were included. If a segment was lengthened, the new attributes (including revised length) of the segment were recorded for follow up audits.

### Data collection

**Fig. 2:**
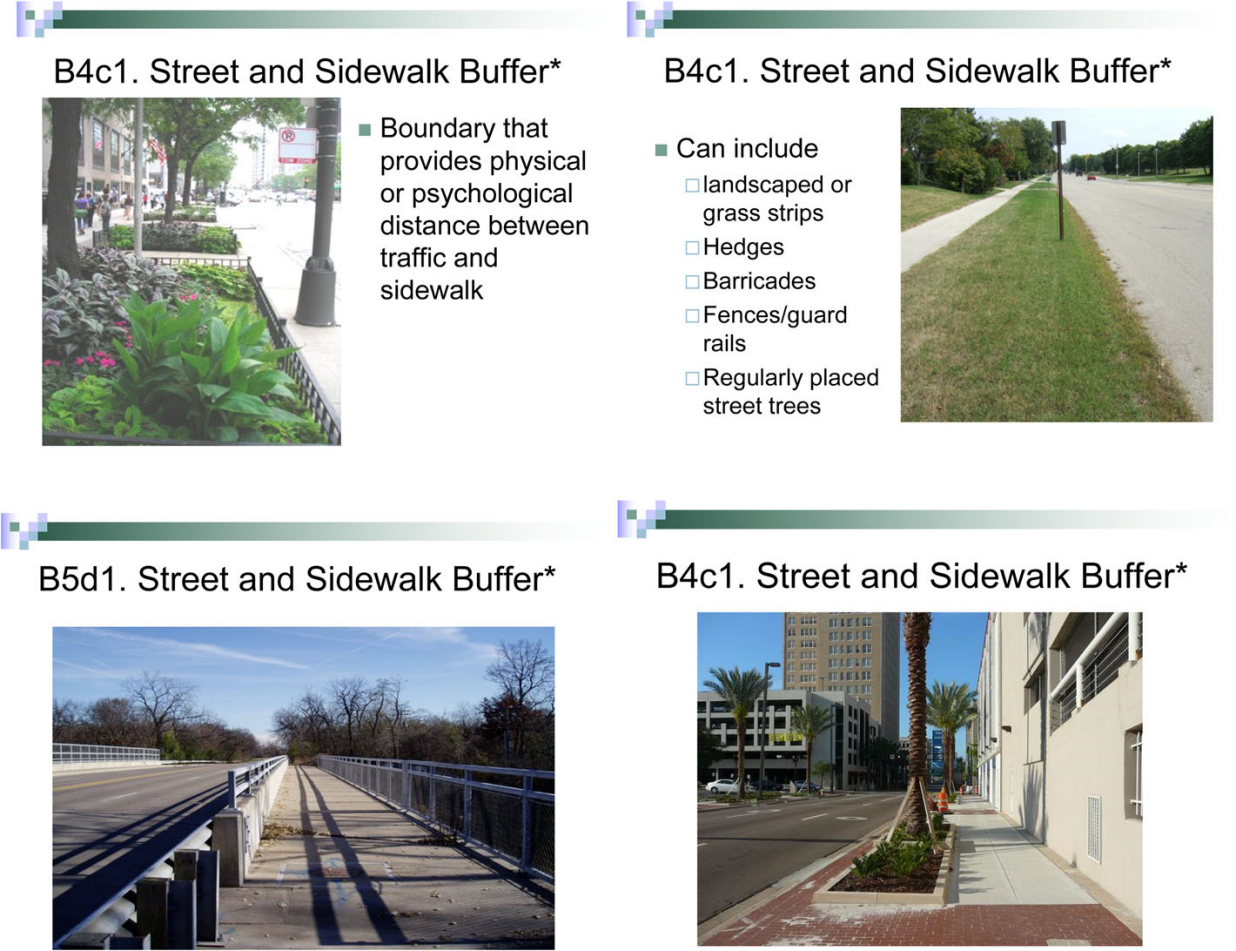
Example of PHRESH SSA classroom training slides. Source: Authors’ own. Legend: Street and Sidewalk Buffer: Refers to a boundary that provides physical or psychological distance between traffic and sidewalk. A street or sidewalk buffer can include landscaped or grass strips, hedges, barricades, fences/guard rails and regularly placed street trees

**Fig. 3:**
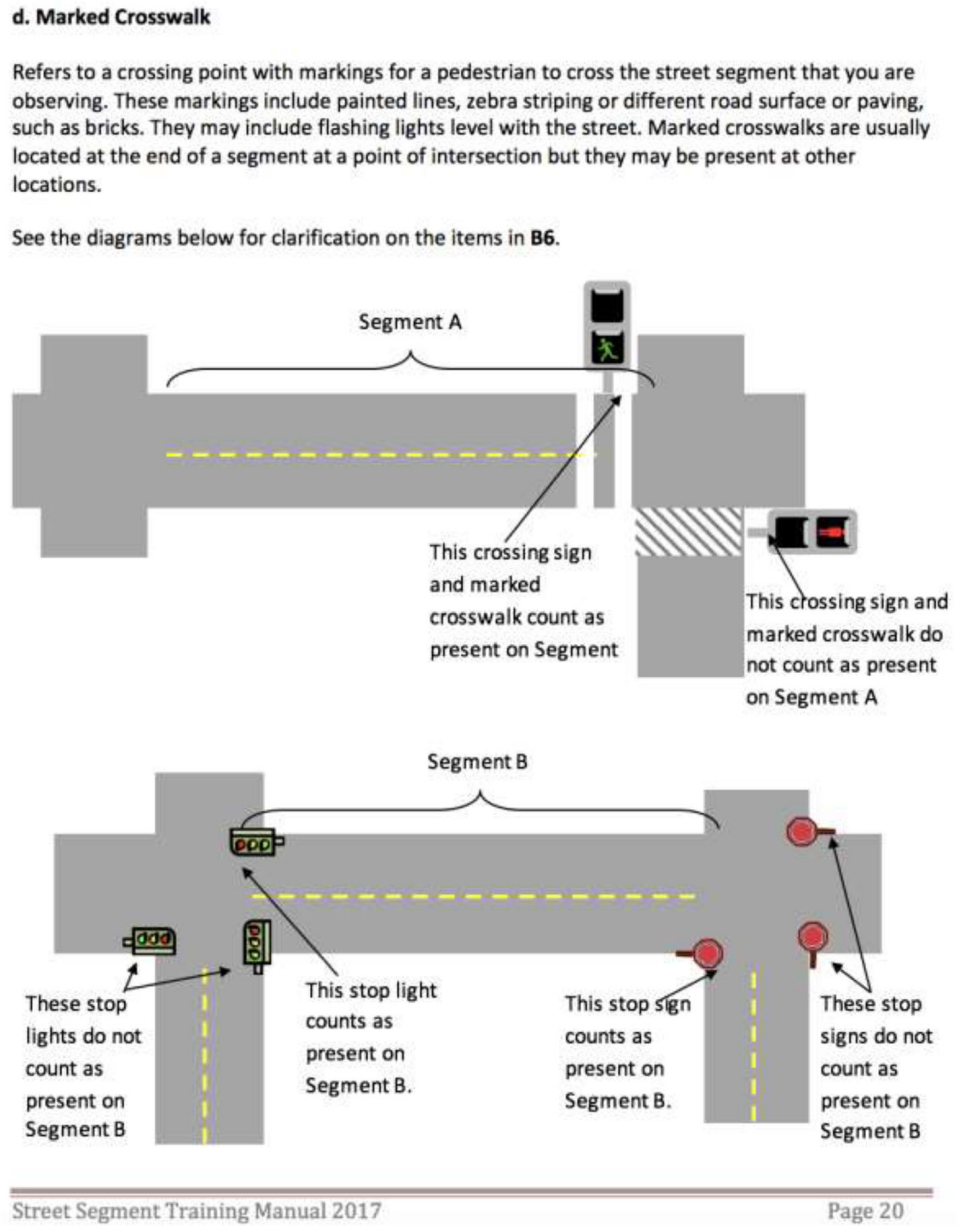
Example of PHRESH SSA Training Manual. Source: Authors’ own. Legend: Marked Crosswalk: Refers to a crossing point with markings for a pedestrian to cross the street segment that you are observing. These markings include painted lines, zebra striping or different road surface or paving, such as bricks. They may include flashing lights level with the street. Marked crosswalks are usually located at the end of a segment at a point of intersection but they may be present at other locations

All data collectors were community members familiar with the neighborhoods, and some of the data collectors participated in two waves (2015, 2017) of data collection. Training was conducted by an experienced trainer and consisted of three parts: (i) in-class presentations including examples and photographs (Fig. 2) with discussions about highlighted characteristics to look for; (ii) field practice on ‘live’ street segments around the training site; and (iii) a certification exercise where the data collectors and the trainer independently rated the same street segment, and compared ratings to test the data collector’s understanding of the tool, observation skills, and data recording technique. Data collectors were given a comprehensive manual with the safety protocol and detailed description of audit tool items accompanied with photo examples (Fig. 3), and a summary sheet responding to common questions asked (e.g. FAQ). Each street segment was audited by a team of two data collectors (hereafter, DC pair), which is shown to improve reliability of ratings [41]. The DC pair walked the street segments together and made a single joint rating for each item, with discussions to resolve disagreements about proposed ratings in real time. A field coordinator oversaw data collection and assigned data collectors to street segments using maps. In each year, audits were conducted between August and October.

### Reliability testing

A random sub-sample of the full sample of street segments, selected for direct observation, was subject to reliability testing (*n* = 60 in 2012, 2015; *n* = 100 in 2017). We drew a sub-sample of about 10% because it was considered reasonable from both a cost and calculation standpoint. While there were not enough segments in the sub-sample to test reliability in the separate neighborhoods, we were able to look at overall reliability if we pooled them together. Each segment in the reliability sub-sample was audited twice within a one-week period. Different DC pairs conducted the two ratings, so that no individual rated the same segment twice. The two ratings were also matched on day and time in 2017 because these factors were considered important for the new physical and social disorder items (see Table 1) added to the 2017 audit tool. Our reliability statistics were chosen to accommodate the response categories used in the SSA tool. About half the items had three response categories (“neither”, “either”, “both sides of the street”), while the rest were mostly binary noting whether a feature was present or absent in that street segment. A few items (e.g. physical disorder) had more than three response categories (e.g. none, a few [1–3], some [4–6], a lot (7 or more)).

**Fig. 4:**
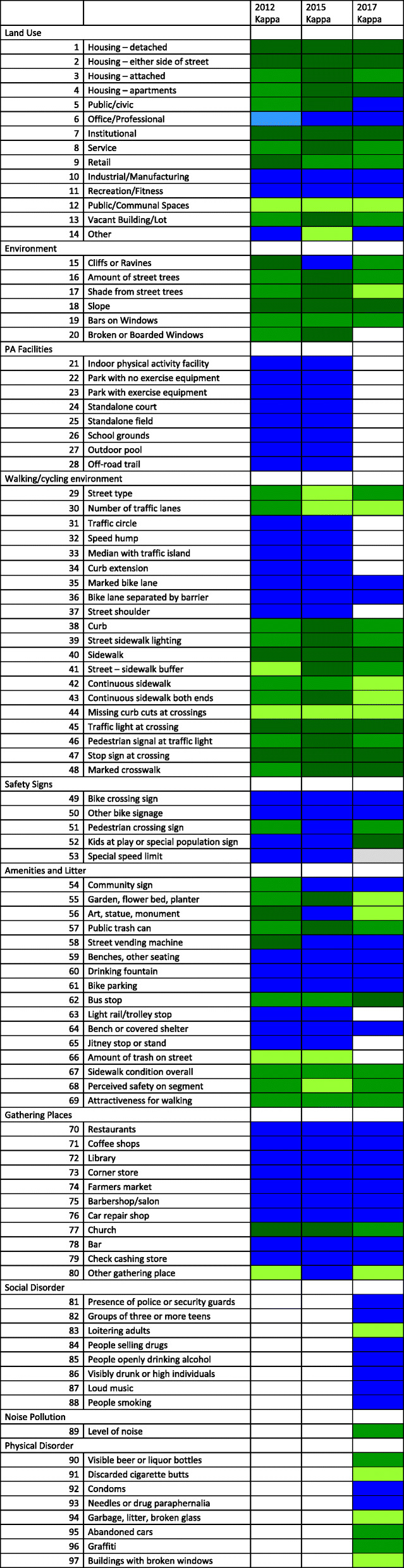
Reliability of Street Segment Audit (SSA) Items. Source: Author’s Calculations. Legend: Krippendorff’s alpha (KA) in green or Percent inter-observer agreement (PO) in blue are displayed. Color-coding show levels of agreement (low, medium or high). While KA is more rigorous, when the distribution of responses for any item is skewed (i.e. a single response category with prevalence > 95%), we cannot obtain stable estimates of the KA statistic. Therefore, we report the PO statistic for these items. Also, “na” indicates that the item was not assessed in that data collection year

Reliability analysis included calculation of prevalence, percentage inter-observer agreement (hereafter, PO) [55, 56] and krippendorff’s alpha (hereafter, KA) [57–60]. Reliability statistics including KA are sensitive to base or prevalence rates. Therefore, while the KA is more rigorous and indicates whether agreement exceeded chance levels, we computed the PO statistic as a supplemental index of interrater reliability for all items. PO indicates the proportion of street segments where DC pairs were in exact agreement (e.g. both ratings were “no” for the same street segment). For Fig. 4, we used the following classification for PO: PO > 90% indicates excellent agreement, PO between 75 and 90% indicates good agreement, and PO < 75% combines moderate and fair to poor agreement [61, 62]. Consistent with prior research, KA > .75 indicates excellent agreement, KA between .40 and .75 indicates intermediate to good agreement, and KA < .40 indicates poor agreement [63]. The reliability statistics can tell us whether an audit tool item has good to excellent agreement at a *single* time point. On the other hand, items with good to excellent agreement at *every* timepoint demonstrate stability, making them appropriate to detect change.

## Results

KA or PO statistics, with color-coding to indicate level of agreement, are displayed in Fig. 4. For most items, we report KA; where items are very common or rare, we report PO. In 2012, 93.8% of items had excellent (62.5%) or good (31.3%) agreement. In 2015, 91.3% of items had excellent (83.8%) or good (7.5%) agreement. In 2017, 83.5% of items had excellent (55.7%) or good (27.8%) agreement. When assessing stability across waves, 81.4% (79 out of 97) of items had good to excellent agreement at every timepoint, making them sufficiently reliable to detect change. Prevalence statistics for individual items are shown in supplemental Table 1.

Twelve of 14 Land use mix items had good to excellent agreement while two items (public/communal spaces, other land use) had poor agreement at all waves. Five out of 6 Environment items had good to excellent agreement across waves, while one item (“do trees shade sidewalk?”) had poor agreement at one of the three waves. Inspection of the individual raters’ responses suggests that raters seemed to have difficulty in choosing “some” versus “many” as a response. For all 8 items in the PA facility category, there was uniformly excellent agreement at each wave.

There were 20 items in the Walking/Cycling environment category. Within the sub-category “*Intersection and Crossing*” including four items (traffic light, pedestrian signal at traffic light, stop sign, marked crosswalk), all had good to excellent agreement at every wave. Of the 8 items in the sub-category “*Street features*”, four showed good to excellent agreement at every wave. Another three items (“street and sidewalk buffer”, “continuous sidewalk”, “sidewalk continuous at both ends between segments) showed poor agreement at one of the waves, while a fourth item (“curb cuts or ramps missing at crossing points”) exhibited consistently poor agreement at every wave. The four items in the sub-category “*Traffic features*” (“traffic circle/roundabout”, “speed hump/table”, “median with traffic island”, “curb extension/bulb-out”) and the two cycling environment items demonstrated good to excellent agreement at every wave. The other two items in Walking/Cycling environment (street type, number of traffic lanes), showed poor agreement at either one or two of the timepoints.

There were five items in the Safety signs category; all were reliably assessed at every wave. 12 out of 16 Amenities and litter items had good to excellent agreement at every wave. Two items (“art or monument”, “garden bed/planter”) showed poor agreement at one of the three waves, while a third item (“amount of trash/litter on street”) showed low agreement at every wave. Of the two more general assessments made by raters (“perceived safety”, “attractiveness of segment for walking”), only one (“perceived safety”) had poor agreement in one wave. Also, PO was excellent for 7 of the 8 items in the Physical activity facility category, and poor for 1 item (“other gathering place”) at two of the three waves.

For 17 items in three categories, we cannot assess agreement at multiple time points because they were only measured in 2017. A single, ordinal item in Noise pollution (with 4 response categories: “no”, “a little”, “some” or “a lot of pollution”) demonstrated good agreement. Seven of the 8 Social disorder items had excellent agreement (PO statistic > 90%) while one item (“adults loitering, congregating, or hanging out”) had poor agreement (PO < 75%). Three of the 8 Physical disorder items (“discarded cigarette butts”, “garbage, litter, broken glass”, “buildings with broken windows”) had low agreement while the other five had good or excellent agreement.

## Discussion

PHRESH is an ongoing study of two low-income and predominantly African American urban communities in Pittsburgh, PA. To assess whether neighborhood-level changes impact residents’ health and well-being, diet, exercise, sleep, heart, and cognitive health, we conducted three assessments of the physical and social environment in the two neighborhoods over a period of five years (2012–2017). The purpose of the parent study is to identify correlates of, and the extent to which neighborhood-level changes, affected obesogenic behaviors such as physical activity, sleep, and heart health. In this paper, we have described our implementation methods, lessons learned, and results from repeated reliability testing of the audit tool (comprised of a standard set of items) to understand if there is stability across time to detect change in the environment over a period of five years. These are offered to inform the design and interpretation of future longitudinal studies of the physical and social environment.

Representative sampling was a critical step. Previous work had demonstrated that a 25% sample of residential street segments produced valid estimates of the built environment [54]. When assessing neighborhood-level change, one difficulty is that these changes can modify the underlying street network. Our experience suggests that secondary sources of data may include non-negligible errors potentially due to delays in updating secondary databases. Whenever feasible (e.g. in a compact environment), we recommend careful verification of available listings of neighborhood street segments to ensure high accuracy. Also, it is necessary to update the street network at each assessment wave to capture the degree of change in the street network. To reflect actual changes in the street network, we carefully identified and sampled new street segments at each wave. When sampling new segments, systematic rules are needed. For instance, when an entire street segment was demolished, should the replacement come from the same geographic area or be sampled entirely at random? Should a newly bisected street count as two new streets, or as the same street segment from a prior wave? A changing street network meant that segment-level panel analysis was difficult; instead, it was more reasonable to identify a stable unit of analysis (e.g. a residential buffer for each study participant) to assess change.

We integrated a community engaged research framework to ensure the longevity and acceptance of PHRESH within the study communities [43]. Our data collectors were recruited from the community, and some of the data collectors were retained across waves. However, we were not able to assess any such effects with our data. Nevertheless, thorough and consistent training of data collectors at each wave was a central feature of this work. Training at each wave employed the same methods and trainer to avoid systematic biases in ratings across waves. During training, it was important to balance classroom learning with ‘live’ practice. In the classroom, the use of visuals (e.g. photographs) worked well. Field practice focused on individual sections of the audit tool and presented a variety of observations. We budgeted extra time to allow data collectors to discuss questions/situations with the trainer. Thus, the training schedule needed to be flexible to allow extra time for hard-to-assess items. Furthermore, we found field practice to be the most valuable part of training. When recruiting data collectors, attention to detail was an important individual trait.

Assessment of (inter-rater) reliability of individual SSA items, using a sub-sample of segments, helped identify items that performed well at a single timepoint, and across time. A majority of SSA items (81%) had high reliability. Low agreement indicated items that were difficult to rate objectively or with a single observation. For example, “amount of litter” or “adults loitering, congregating or hanging out” may vary even over a short window of time (e.g. a few hours or a day). In the case of trash, we re-assessed agreement for a small subset of street segments in the reliability study where two observations were conducted within hours of each other. However, the agreement for trash or litter did not improve. Items with substantial temporal variation may require multiple ratings (> 2) to accurately capture the average or mean rating. Certain items (e.g. perceived safety) were inherently subject to interviewer interpretation, and demonstrated lower agreement, as expected. Few neighborhood features were not easily visible across an entire street segment (e.g. bar on a single window, cigarette butts on the ground; garden bed/planter), or difficult to assess from the outside (e.g. public/communal space, vacant building) as was necessary according to the audit protocol.

Given these study findings, we can suggest the types of items that may be able to capture change. Consistent with previous research, more subjective measures are less reliable than more objective (observable) ones [41]; dichotomous ratings have higher reliability than ordinal response scales (although a greater number of response categories may be valuable for providing finer distinctions). Large, visible items (e.g. buildings, traffic signs) were consistently reliable. While sidewalks are an important feature of the walking environment, sidewalk conditions may change quickly over a city block, making it challenging to rate consistently. Also, rare/low prevalence features (see supplemental Table 1) did not lend themselves well to KA testing. For example, the only gathering places in these neighborhoods with prevalence above 5% were churches. If low prevalence items were readily identified, the PO statistic showed consistency in endorsing their absence.

While some features of the environment may change, there were features that are time invariant. Yet, when we compared slope (“flat”, “slight hill”, “steep hill”) across years for a sub-group of street segments with three years of complete data, 22% of the segments had different values although slope is unlikely to change. Also, 10% of street segments were endorsed as having art/monument in 2012, while only 2% of segments had art/monument three years later (2015.) which may point to confusion over what constitutes art. Therefore, we recommend the use of SSA items with consistently good to excellent agreement across repeat assessments to detect real change. Future studies may be able to further improve the measurement of less reliable items through detailed and intensive training or procedures (e.g., mapping out a visual area into a grid to more systematically inspect for broken windows), clearer rules and examples for determining whether something is a communal space, or by the addition of a “cannot determine” category to the form. Even subjective ratings may be improved if anchored through training or explicit item instructions (e.g. 1 = a place where you would not feel physically at risk of violence from another person if walking alone in daylight, etc.), and by use of multiple raters to reduce individual rater idiosyncrasies.

In our knowledge, this article is the first to conduct repeated assessments of the built and social environment to assess change. We found the PHRESH study’s SSA tool to be reliable and practical to implement, with an average of 13 min required per street segment, that data collectors found easy to use. The audit tool provided rich and detailed data on environmental features, and change over time, which is important for the exploration of cross-sectional and longitudinal relationships between neighborhood features and health outcomes. The compact nature of our study neighborhoods suggests a need to test this audit tool in neighborhoods with greater variation, as certain items exhibited low or zero prevalence in the study neighborhoods. Future research might want to evaluate reliability separately if comparing change across neighborhoods for a natural experiment or intervention study. Our sample sizes for the reliability sub-sample were only sufficient to assess overall reliability by pooling sample across neighborhoods. Future study design can consider sample allocation so that the two neighborhoods (with and without intervention) are assessed with equal reliability. Also, additional steps are necessary to develop and validate summary measures or indices that capture meaningful constructs (e.g. walkability, incivilities) that may be predictors of health outcomes. If valid indices of environmental features can be derived, they will be useful in guiding public policy and urban planning in the redesign of built environments to promote health.

## Conclusion

This paper presents lessons learned from repeat administrations of a comprehensive audit tool for direct observation of the environment. Longitudinal assessments required consistency of methods and data collector training to minimize systematic differences across time. Inter-rater reliability testing conducted at each time point suggested that most items were consistently reliable and were useful to assess changes in the environment. Typically, items with poor reliability were either difficult to rate or subjective in nature, making them less useful to detect real change over time. The PHRESH-SSA tool proved to be a generally reliable and practical instrument for collecting data that trained observers found easy to use.

## Supplementary information

**Additional file 1.**

## Data Availability

The datasets used and/or analyzed during the current study are available from the corresponding author on reasonable request.

## Abbreviations

BTG-COMP: Bridging the Gap/Community Obesity Measures Project
GSV: Google Street View
GIS: Geographic information systems
SSA: Street Segment Audit Tool
SPACES: Systematic Pedestrian and Cycling Environmental Scan
SLU: St. Louis Analytic Audit Tool and Checklist
PEDS: Pedestrian Environment Data Scan
PHRESH: Pittsburgh Hill/Homewood Research on Neighborhood Change and Health
KA: Krippendorff’s alpha
PO: Percent observer agreement
ESRI: Environmental Systems Research Institute
